# Personalized Tibial Component Placement in Medial Unicompartmental Knee Arthroplasty: Surgical Technique and Rationale

**DOI:** 10.3390/jcm15103797

**Published:** 2026-05-14

**Authors:** Paolo Queirazza, Marco Minelli, Francesco Cacace, Elizaveta Kon, Enrico Arnaldi, Marco Basso

**Affiliations:** 1 IRCCS Humanitas Research Hospital, Via Manzoni 56, Rozzano, 20089 Milan, Italy; paolo.queirazza@humanitas.it (P.Q.); francesco.cacace@humanitas.it (F.C.); elizaveta.kon@humanitas.it (E.K.); enrico.arnaldi@humanitas.it (E.A.); marco.basso@humanitas.it (M.B.); 2 Department of Biomedical Sciences, Humanitas University, Via Rita Levi Montalcini 4, Pieve Emanuele, 20072 Milan, Italy

**Keywords:** unicompartmental, knee, arthroplasty, personalized, alignment

## Abstract

Unicompartmental knee arthroplasty (UKA) is an effective treatment for anteromedial osteoarthritis in carefully selected patients. Increasing attention has recently been directed toward restoration of pre-arthritic coronal alignment, supported by the use of the arithmetic hip–knee–ankle angle (aHKA) to estimate constitutional lower limb alignment. In medial UKA, kinematic alignment principles derived from the original technique described by Cartier et al. may help to reproduce native joint-line orientation while preserving physiological soft-tissue balance. This technical note details the indications, preoperative assessment, planning strategy, and operative steps of the procedure. Preoperative long-leg weight-bearing radiographs are used to estimate constitutional alignment through the aHKA and to plan the coronal inclination of the tibial cut. Intraoperatively, the distal position of the extramedullary guide is reproduced according to the preoperative planning in order to restore the native inclination of the medial tibial plateau. The sagittal tibial cut, posterior tibial slope, distal femoral cut, component sizing, gap assessment, and cementation technique are described, with emphasis on anatomical landmarks and technical pearls to improve reproducibility. The described technique provides a practical method for approximating constitutional coronal alignment in medial UKA without the use of robotic or navigated systems. The key feature of the procedure is accurate planning and execution of the tibial cut in both the coronal and sagittal planes in order to reproduce native joint-line orientation and preserve appropriate ligament balance.

## 1. Introduction

Anteromedial osteoarthritis (AMOA) of the knee is a common cause of pain and functional limitation in patients with a correctable varus deformity and an intact anterior cruciate ligament (ACL), since complete ACL disruption has been associated with more advanced knee arthritis and a pattern of disease that is less suitable for this procedure [1,2]. In appropriately selected patients, unicompartmental knee arthroplasty (UKA) is an effective treatment option, with lower perioperative morbidity than total knee arthroplasty and restoration of knee kinematics closer to those of the native joint [3,4,5].

The introduction of the arithmetic hip–knee–ankle angle (aHKA), which has proven to be a reliable method for estimating constitutional lower limb alignment in arthritic knees, has renewed interest in restoring pre-arthritic coronal alignment [6,7].

Although the conventional UKA technique has largely been derived from mechanical alignment principles, several studies suggest that a kinematic alignment-inspired approach may improve functional outcomes and reduce stress at the tibial component [8,9,10].

Thirty years ago, Cartier et al. described a technique for UKA component positioning based on restoration of native joint-line orientation. In this technique, the tibial component is positioned perpendicular to the proximal tibial metaphyseal–epiphyseal axis, and the femoral component is aligned perpendicular to the tibial cut [11].

The aim of this article is to describe our surgical technique, performed with conventional instrumentation, to achieve coronal kinematic alignment in medial UKA according to the principles originally described by Cartier et al.

## 2. Surgical Technique

### 2.1. Clinical Examination

The indications and contraindications for unicompartmental knee arthroplasty are summarized in Table 1. Clinical assessment should specifically evaluate for flexion contracture and non-correctable varus deformity, which represent major contraindications to the procedure. Functional integrity of the ACL must also be confirmed clinically to exclude anteroposterior instability.

### 2.2. Preoperative Planning

**Figure 1:**
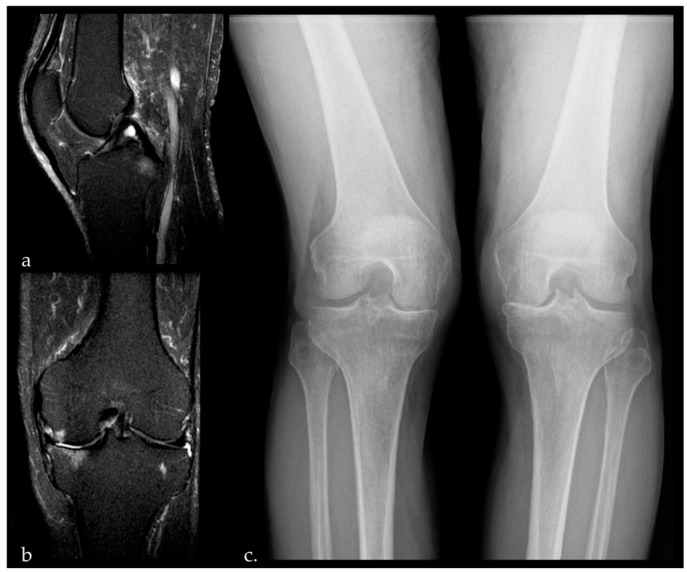
Preoperative knee MRI confirming intact ACL with preserved tension (**a**) and absence of significant chondral or meniscal pathology in the lateral compartment in an 82-year-old male patient (**b**). Rosenberg view demonstrating bone-on-bone anteromedial osteoarthritis (**c**).

Preoperative imaging includes anteroposterior, lateral, axial, and Rosenberg radiographs of the knee, together with full-length weight-bearing radiographs of both lower limbs (Figure 1). Magnetic resonance imaging may be useful as a second-line investigation to exclude relevant chondral lesions or meniscal pathology in the lateral compartment and to confirm ACL integrity and AMOA.

Pre-arthritic coronal alignment was estimated on long-leg radiographs using the arithmetic hip–knee–ankle angle (aHKA), calculated as the difference between the medial proximal tibial angle (mMPTA) and the mechanical lateral distal femoral angle (mLDFA): *aHKA* = *mMPTA* − *mLDFA.* In this study, negative values indicate constitutional varus alignment and positive values indicate constitutional valgus alignment. Postoperative coronal limb alignment was assessed using the hip–knee–ankle angle (HKA). Because aHKA represents an estimate of constitutional alignment and postoperative HKA represents the achieved postoperative limb alignment, the two values are not interchangeable but are compared to determine how closely the reconstruction approximates the predicted pre-arthritic alignment. Restoration of constitutional alignment was considered satisfactory when the postoperative HKA fell within ±3° of the preoperative aHKA estimate.

**Figure 2:**
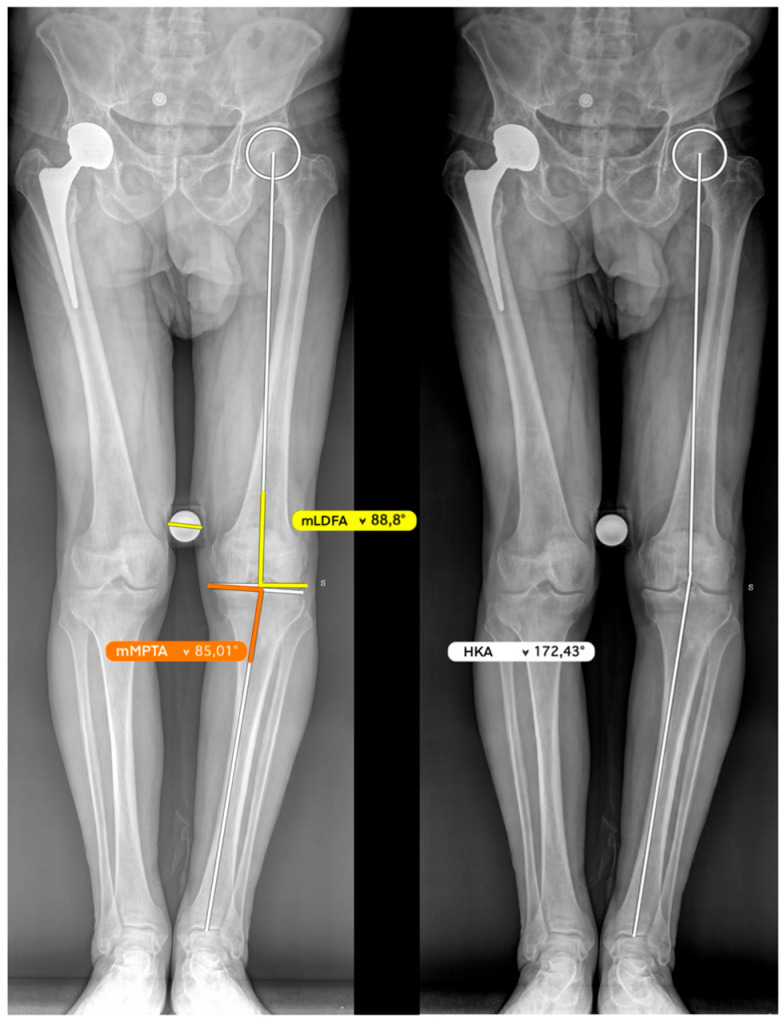
Weight-bearing full-length lower limb radiograph used for preoperative planning. The medial proximal tibial angle (mMPTA) is measured between the tibial mechanical axis—drawn from the center of the tibial plateau to the center of the tibiotalar joint—and the medial tibial plateau surface. The mechanical lateral distal femoral angle (mLDFA) is measured between the femoral mechanical axis—from the center of the femoral head to the center of the knee—and the lateral distal femoral articular surface. The anatomical hip–knee–ankle angle (aHKA) is calculated as the difference between the preoperative mMPTA and the mLDFA, reflecting the overall coronal alignment of the lower limb (in this case aHKA = 85° − 89° = −4°).

Using preoperative planning software, the radiograph is calibrated against a known reference. A line perpendicular to the tangent of the medial tibial plateau is then drawn to simulate the extramedullary rod used intraoperatively during the sagittal cut. The vertex of this angle is positioned just below the medial tibial spine, at approximately the site where the first fixation pin for the cutting guide will be inserted. The distal position of this line relative to the center of the ankle is then measured so that it can be reproduced intraoperatively (Figure 2).

### 2.3. Operative Technique

The critical steps, together with pearls and pitfalls, are summarized in Table 2.

The patient is positioned supine on the operating table. A lateral support is placed proximally at the level of the tourniquet. Two foot supports are positioned distally to stabilize the knee at 90° and 120° of flexion.

**Figure 3:**
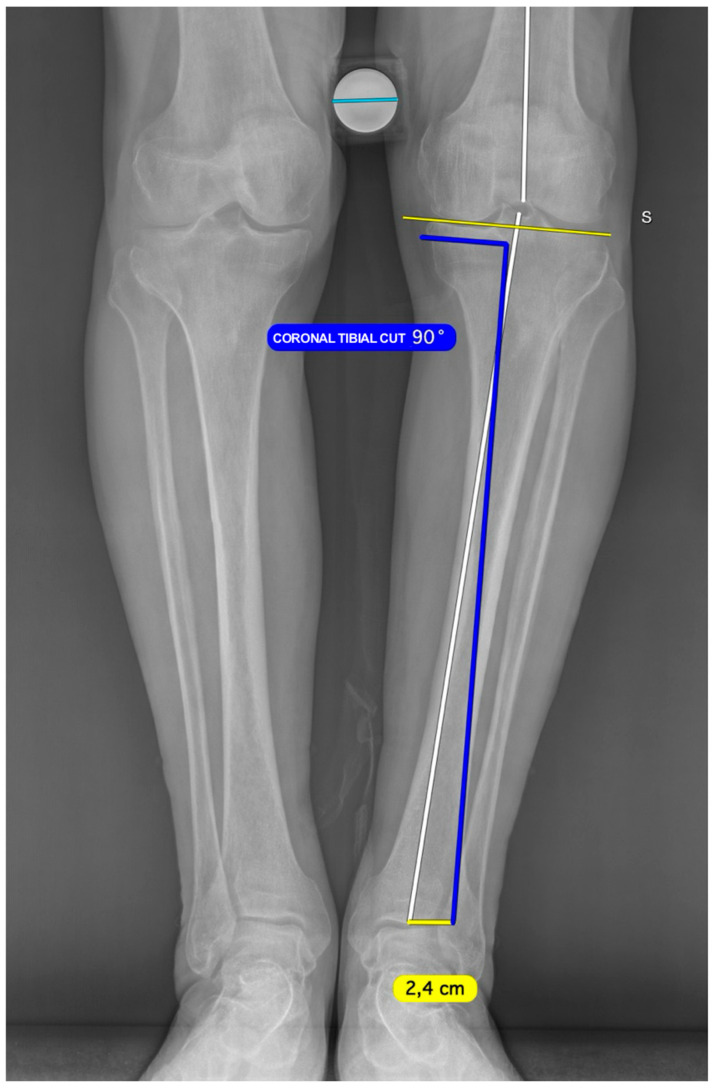
Weight-bearing full-length lower limb radiograph illustrating preoperative planning for the coronal tibial cut. A tangent line is drawn along the lateral tibial plateau surface, serving as the reference for the native joint-line orientation. A 90° angle is constructed perpendicular to this tangent, representing the intended inclination of the tibial cutting guide in the coronal plane. The apex of this right angle is positioned at the level of the anticipated proximal pin insertion site. The distance at which the perpendicular line passes distal to the center of the tibiotalar joint is measured and recorded, as this value is replicated intraoperatively to ensure accurate and reproducible positioning of the cutting guide.

The medial and lateral femoral epicondyles, tibial tuberosity, tibial crest, and center of the tibiotalar joint are identified. The point at which the extramedullary rod should pass distally is marked on the skin according to the distance measured during preoperative planning from the center of the ankle joint (Figure 3).

With the knee flexed to 90°, a 10 to 12 cm skin incision is made just medial to the patellar midline. The subcutaneous tissue is dissected to the joint capsule, and a medial mini-midvastus arthrotomy is performed, preserving 5 to 10 mm of capsular tissue attached to the patellar edge to facilitate closure.

To improve visualization of the medial compartment, a limited amount of prepatellar fat is removed, the anterior third of the medial meniscus is excised, and the medial capsule is progressively released until the osteophyte at the medial tibial plateau becomes visible. Care must be taken to preserve the function of the superficial medial collateral ligament (MCL). Osteophytes from the intercondylar notch and medial femoral condyle are then removed. Intra-articular assessment should confirm that the ACL is functionally intact, the medial compartment shows advanced anteromedial osteoarthritis, patellofemoral degeneration is limited, and the lateral compartment is preserved.

The tibial cut is the key step in reproducing alignment close to the patient’s constitutional pre-arthritic alignment. It includes a sagittal cut, which determines rotational positioning of the tibial component, and a coronal cut, which determines varus–valgus orientation, posterior slope, and resection level.

With the knee flexed and the ankle maintained in neutral rotation, the sagittal cut is oriented close to the ACL fibers and directed toward the anterior superior iliac spine (ASIS). These steps help achieve reproducible external rotation of the tibial component of approximately 4° to 5°. Osteophytes in the intercondylar notch must be adequately removed so that the sagittal cut can be made parallel to the medial femoral condyle and as lateral as possible. The saw blade is then left in place to stabilize the saw trajectory during the coronal cut and to prevent undermining of the tibial eminence.

**Figure 4:**
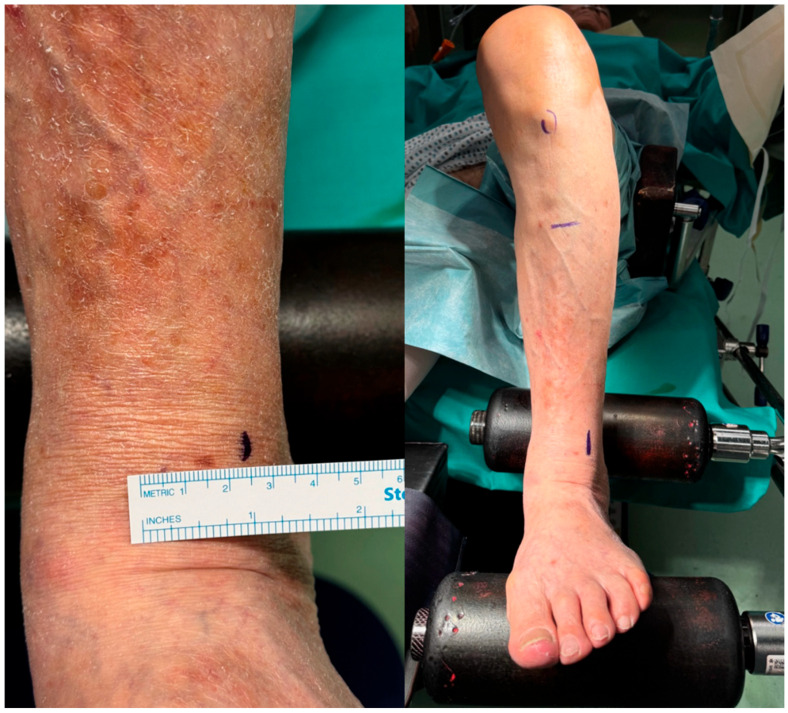
Preoperative skin markings reproducing the distance from the center of the tibiotalar joint, as determined during preoperative planning, to guide the distal positioning of the coronal tibial cutting guide intraoperatively.

The knee is flexed to approximately 120°. The cutting guide is fixed with a pin about 1 cm below the medial intercondylar eminence after assessment of resection depth using a 2 mm or 4 mm stylus, depending on the degree of cartilage wear. The extramedullary rod is then aligned distally with the skin mark corresponding to the preoperative plan (Figure 4). In this way, the resection reproduces the coronal inclination of the native medial tibial plateau.

Posterior slope is assessed using an alignment guide placed in the cutting block. To reproduce the native posterior slope regardless of osteoarthritis severity, the guide should be parallel to the insertion line of the deep MCL. This line, connecting the anterior and posterior insertion points of the deep MCL, represents a consistent anatomical reference. After final verification of alignment, the cutting guide is secured with a second pin and the coronal cut is performed while protecting the MCL with a retractor.

The distal femoral cut determines coronal alignment of the femoral component. It is performed in extension with an 8 mm spacer inserted into the medial compartment, ensuring that the spacer lies flat on the tibial cut and contacts the distal femur. A line is then drawn from the midpoint of the anterior cortex of the medial femoral condyle, perpendicular to the tibial cut, to define the center line of the femoral component. The distal femur is first cut in extension using the cutting guide and then the cut is completed freehand in flexion to increase distance from the posterior neurovascular structures.

Before assessing balance, the remaining medial meniscus is removed and any incomplete tibial cuts are completed. Appropriate balance is achieved when flexion and extension gaps are equivalent and there is approximately 2 mm of laxity under valgus stress. Overall limb alignment is then checked before completing the femoral preparation.

With the knee flexed, the final femoral cutting guide is positioned parallel to the previously marked center line of the femoral component and flush with the distal femoral cut surface. If a uniform gap of approximately 2 mm is present between the anterior femoral cartilage and the anterior flange of the guide, the size is considered appropriate. The guide should be lateralized to align the femoral component with the lateral margin of the medial femoral condyle and to avoid impingement in the intercondylar notch.

**Figure 5:**
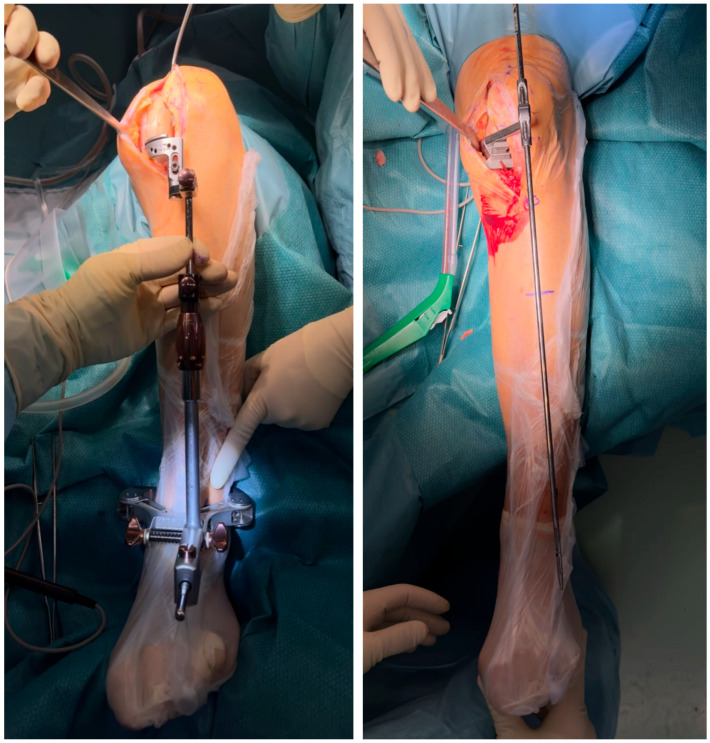
Intraoperative alignment assessment performed before and after the coronal tibial and femoral cuts, confirming the accuracy of component positioning relative to the preoperative plan.

Final positioning is confirmed by checking that the posterior margin of the guide is parallel to the tibial resection (Figure 5). The guide is then fixed, and the remaining cuts and peg holes are prepared.

To determine mediolateral and anteroposterior dimensions, the tibial sizing guide is placed in direct contact with the medial cortex. Overhang beyond the cortical margin should be avoided to ensure even load distribution. If required, further lateralization of the sagittal tibial resection should be considered. If this is not possible, a smaller tibial component should be selected. Once appropriate sizing has been confirmed, the tibial plateau is prepared using the implant-specific instruments.

After completion of bone preparation, trial femoral, tibial, and polyethylene components are inserted. The knee is taken through a full range of motion to assess stability, congruency, and component tracking.

Gap balance in flexion and extension is reassessed using a 2 mm feeler gauge: the 2 mm gauge should pass with slight resistance, whereas a 3 mm gauge should be difficult to insert. This tactile feedback indicates appropriate ligament tension.

Cement is applied to the definitive components. In the presence of sclerotic bone, superficial drill holes may be considered to improve cement penetration. A thin layer of cement is applied to the cut tibial surface and pressurized manually using a broad flat instrument in a posteroanterior direction. The tibial component is inserted manually from posterior to anterior with gentle pressure to encourage anterior extrusion of excess cement and reduce the risk of posterior cement retention. Forceful impaction should be avoided to minimize the risk of tibial plateau fracture. Excess tibial cement must be removed before femoral cementation.

The femoral surface is prepared in a similar manner by drying the bone and pressurizing cement into the peg holes. With the knee in maximal flexion, the posterior peg is inserted first, followed by the anterior peg. The femoral component is then gently impacted and excess cement is removed. A trial insert is placed, combined with a 2 mm spacer, to obtain adequate compression during cement polymerization.

**Figure 6:**
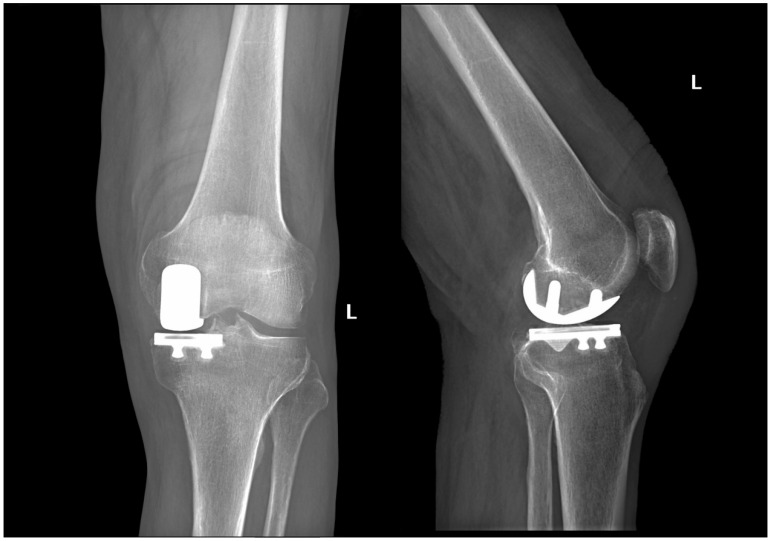
Postoperative radiographs at 45 days from surgery showing accurate prosthetic component positioning, joint-line restoration, and reciprocal alignment between the tibial and femoral components.

**Figure 7:**
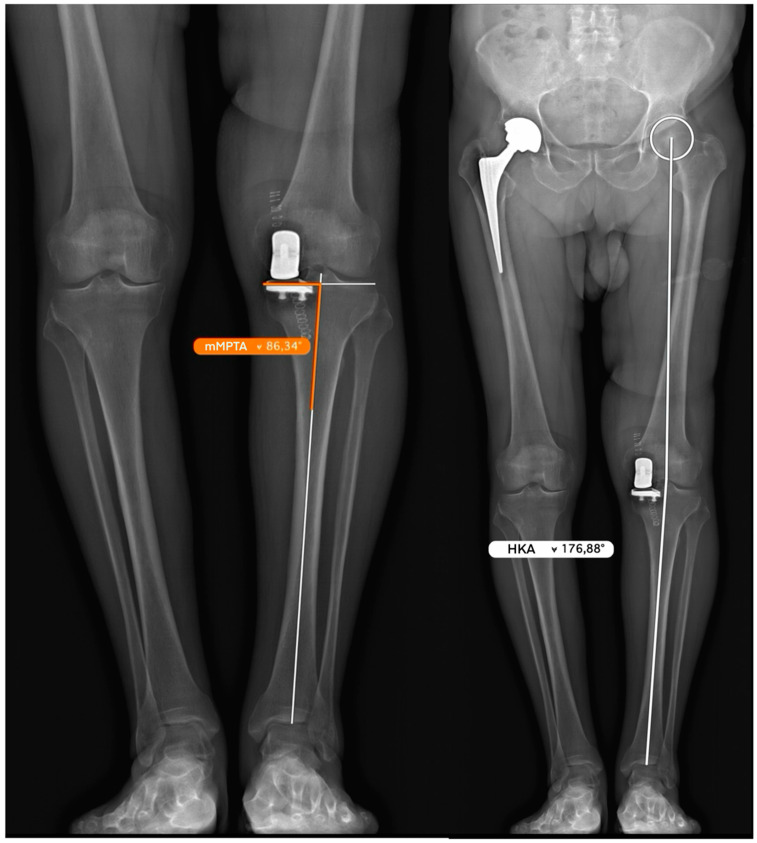
Full-length postoperative radiograph confirming restoration of the patient’s constitutional lower limb alignment: the measured mMPTA reflects the executed coronal tibial cut, while the postoperative HKA matches the preoperatively determined aHKA (aHKA = −4°).

After cement polymerization, the definitive polyethylene insert is implanted following thorough irrigation, hemostasis, and removal of any remaining cement (Figure 6 and Figure 7). The wound is then closed in layers, as shown in Table 2.

## 3. Discussion

The purpose of this technical note is to describe a reproducible method for component positioning in medial UKA according to the alignment principles originally described by Cartier et al. using conventional instrumentation and radiograph-based preoperative planning. The recent literature suggests that slight varus positioning of the tibial component may be associated with favorable clinical outcomes and improved load distribution across the tibial implant [8,12].

The central feature of the present technique is therefore the coronal inclination of the tibial cut, which is intended to restore the patient’s constitutional joint-line orientation by aligning the tibial component perpendicular to the tibial metaphyseal–epiphyseal axis. To achieve this, preoperative planning is performed on full-length weight-bearing radiographs to define both the orientation of the tibial cutting guide and its distal relationship to the center of the ankle. This information can then be reproduced intraoperatively without the need for computer navigation or robotic assistance.

Nevertheless, some borderline cases require particular caution when planning coronal tibial alignment. Several authors have recommended to avoid tibial component positioning beyond 6° of varus, which corresponds to the historical Cartier threshold, and to favor instead a more conservative correction in such cases [13].

Even when instrumentation limits the maximum permitted varus, careful planning remains essential. In particular, the vertex of the preoperative right angle used to simulate the cutting guide must correspond to the expected position of the proximal fixation pin; otherwise, overcorrection or undercorrection may occur.

Posterior tibial slope is another critical parameter in medial UKA because it may influence both postoperative function and implant survival. Excessive slope has been associated with increased postoperative pain and a higher risk of tibial component loosening [14,15]. Reproducing the native posterior slope is conceptually consistent with restoration of pre-disease ligament tension, particularly of the cruciate ligaments, and may reduce the risk of soft-tissue imbalance. In this respect, the deep MCL insertion line described by Parratte et al. provides a useful intraoperative reference because it remains anatomically constant regardless of cartilage wear [16].

Axial alignment of the tibial component, determined by the sagittal tibial cut, also affects component sizing. Adequate removal of intercondylar notch osteophytes is essential before performing the sagittal cut, as residual osteophytes may compromise rotational accuracy. The cut should be directed as laterally as possible, adjacent to the ACL fibers, with a target external rotation of approximately 4° to 5°. This orientation has been reported to improve component fit, preserve surrounding soft tissues, and support satisfactory functional results [17,18].

This technical note has several limitations. First, this manuscript is a technical note describing an operative strategy and does not include clinical, radiographic, or implant survivorship data; therefore, it does not permit conclusions regarding superiority or equivalence relative to conventional mechanically aligned UKA or technology-assisted techniques. Second, although the proposed method is based on preoperative radiographic planning and identifiable intraoperative anatomical landmarks, its accuracy remains dependent on radiograph quality, correct calibration, and surgeon experience, which may affect reproducibility. Third, restoration of constitutional alignment is inferred from the preoperative aHKA and from execution of the planned tibial cut, but this does not necessarily guarantee exact recreation of the patient’s native pre-arthritic joint-line orientation in every case. In addition, this technical note does not include a formal assessment of inter- or intra-observer reliability for preoperative aHKA measurement, nor does it quantify the reproducibility of intraoperative skin marking used to reproduce the planned extramedullary guide position. These aspects would require a dedicated validation study with repeated measurements and prospective reproducibility analysis. Finally, particular caution is required in borderline cases with pronounced varus deformity, especially when preoperative planning suggests a tibial varus near or beyond 6°. In these knees, exact replication of the constitutional joint-line obliquity may expose the implant to a less favorable mechanical environment, and a more conservative correction should be considered. Likewise, severe proximal tibial bone loss, poor cortical support, and markedly reduced bone quality may make a varus tibial resection less safe by reducing the supporting bone beneath the component and increasing fracture risk. For this reason, compromised tibial bone stock should be regarded as a relative contraindication to aggressive personalization of the tibial varus [19,20].

In conclusion, this technique provides a practical method for approximating constitutional coronal alignment in medial UKA using careful radiographic planning and conventional instrumentation alone. It may represent a useful option when robotic or navigated systems are unavailable, although further studies are required to evaluate its accuracy, reproducibility, and clinical outcomes [21,22,23].

## Figures and Tables

**Table 1:** **Indications and contraindications for unicompartmental knee arthroplasty.**

Indications (Essential Criteria)	Contraindications (Absolute/Relative)
Isolated medial compartment OA with bone-on-bone contact	ACL insufficiency
Intact and functional ACL	Inflammatory arthritis
Preserved lateral compartment	Lateral or bicompartmental OA
Correctable varus deformity < 10°	Severe patellofemoral OA
Stable collateral ligaments	Fixed deformity (varus > 10°, valgus > 5°)
Flexion > 100° with flexion contracture < 10°	Flexion contracture ≥ 10–15°
Low-grade patellofemoral wear	

**Table 2:** **Pearls and pitfalls of kinematic alignment in unicompartmental knee arthroplasty.**

	Pearls	Pitfalls
**Bony Landmarks and Markings**	Skin marking to guide the extramedullary rod according to preoperative planning.	Inconsistent skin markings = difficulty in reproducing preoperative planning.
**Sagittal Tibial Cut**	- Cut oriented toward ASIS for correct external rotation of tibial base plate. - Remove osteophytes from the intercondylar notch. - Cut close to the ACL fibers. - Leave the blade in place to guide the coronal cut and avoid undermining the medial tibial spine.	- Excessive medial cut → possible difficulties in tibial sizing. - Excessive or insufficient rotation → ML/AP mismatch.
**Coronal Tibial Cut**	- Align the extramedullary rod with the preoperative skin marking. - Use the insertion line of the deep LCM to reproduce the native slope.	
**Femoral Distal Cut**	- Mark the center line of the femoral component to guide correct rotation. - Complete the posterior cut in flexion to protect the posterior neurovascular structures.	A spacer that is not correctly in contact with the cutting surfaces may cause misalignment and poor reciprocity between the components.
**Femoral Sizing**	- Position the guide parallel to the central femoral axis. - Lateralize the guide to avoid intercondylar conflicts. - Confirm parallelism between the posterior margin of the guide and the tibial resection.	- Non-parallel guidance → limited tibiofemoral congruence. - Excessive anterior impingement → conflict with patella or notch.
**Tibial Sizing**	Lateralize the sagittal resection, if necessary.	ML and AP overhangs (>2 mm) increase the risk of tibial plateau fracture.
**Cementation**	- Press the tibial component from posterior to anterior to minimize posterior cement extrusion. - Position the femoral component in maximum knee flexion, first inserting the posterior peg.	- Excessive percussion → fracture of the tibial plateau. - Posterior cement residues may interfere with the insert or become mobilized.

## Data Availability

The original contributions presented in this study are included in the article. Further inquiries can be directed to the corresponding author.

## References

[1] White S.H., Ludkowski P.F., Goodfellow J.W. (1991). Anteromedial osteoarthritis of the knee. J. Bone Jt. Surg. Br..

[2] Passaretti A., Colò G., Bulgheroni A., Vulcano E., Surace M.F. (2024). Gonarthrosis and ACL lesion: An intraoperative analysis and correlations in patients who underwent total knee arthroplasty. Minerva Orthop..

[3] Kulshrestha V., Datta B., Kumar S., Mittal G. (2017). Outcome of Unicondylar Knee Arthroplasty vs. Total Knee Arthroplasty for Early Medial Compartment Arthritis: A Randomized Study. J. Arthroplast..

[4] Lim J.W., Cousins G.R., Clift B.A., Ridley D., Johnston L.R. (2014). Oxford Unicompartmental Knee Arthroplasty Versus Age and Gender Matched Total Knee Arthroplasty—Functional Outcome and Survivorship Analysis. J. Arthroplast..

[5] Patil S., Colwell C.W.J., Ezzet K.A., D’Lima D.D. (2005). Can Normal Knee Kinematics Be Restored with Unicompartmental Knee Replacement?. JBJS.

[6] Griffiths-Jones W., Chen D.B., Harris I.A., Bellemans J., MacDessi S.J. (2021). Arithmetic hip-knee-ankle angle (aHKA): An algorithm for estimating constitutional lower limb alignment in the arthritic patient population. Bone Jt. Open.

[7] Liu C., Huang C., Suyalatu X., Zhang Q., Zhang Y., Sun W., Guo W., Wang W. (2025). Optimizing uni-compartmental knee arthroplasty: The impact of preoperative planning and arithmetic hip-knee-ankle angle. BMC Musculoskelet. Disord..

[8] Plancher K.D., Brite J.E., Briggs K.K., Petterson S.C. (2022). Pre-Arthritic/Kinematic Alignment in Fixed-Bearing Medial Unicompartmental Knee Arthroplasty Results in Return to Activity at Mean 10-Year Follow-up. JBJS.

[9] Nie Y., Yu Q., Shen B. (2021). Impact of Tibial Component Coronal Alignment on Knee Joint Biomechanics Following Fixed-bearing Unicompartmental Knee Arthroplasty: A Finite Element Analysis. Orthop. Surg..

[10] Dai X., Fang J., Jiang L., Xiong Y., Zhang M., Zhu S. (2018). How does the inclination of the tibial component matter? A three-dimensional finite element analysis of medial mobile-bearing unicompartmental arthroplasty. Knee.

[11] Cartier P., Sanouiller J.L., Grelsamer R.P. (1996). Unicompartmental knee arthroplasty surgery: 10-Year minimum follow-up period. J. Arthroplast..

[12] Vasso M., Del Regno C., D’Amelio A., Viggiano D., Corona K., Panni A.S. (2015). Minor varus alignment provides better results than neutral alignment in medial UKA. Knee.

[13] McEwen P., Omar A., Hiranaka T. (2024). Unicompartmental Knee Arthroplasty: What is the optimal alignment correction to achieve success? The role of kinematic alignment. J. ISAKOS.

[14] Hernigou P., Deschamps G. (2004). Posterior Slope of the Tibial Implant and the Outcome of Unicompartmental Knee Arthroplasty. JBJS.

[15] Chatellard R., Sauleau V., Colmar M., Robert H., Raynaud G., Brilhault J. (2013). Medial unicompartmental knee arthroplasty: Does tibial component position influence clinical outcomes and arthroplasty survival?. Orthop. Traumatol. Surg. Res..

[16] Parratte S., Daxhelet J., Argenson J.N., Batailler C. (2023). The Deep-MCL Line: A Reliable Anatomical Landmark to Optimize the Tibial Cut in UKA. J. Pers. Med..

[17] Iriberri I., Aragón J.F. (2014). Alignment of the tibial component of the unicompartmental knee arthroplasty, assessed in the axial view by CT scan: Does it influence the outcome?. Knee.

[18] Sava M.P., Leica A., Scala I., Beckmann J., Hirschmann M.T. (2023). Significant correlations between postoperative outcomes and various limb and component alignment strategies in medial unicompartmental knee arthroplasty: A systematic review. J. Exp. Orthop..

[19] Yoshikawa R., Hiranaka T., Okamoto K., Fujishiro T., Hida Y., Kamenaga T., Sakai Y. (2020). The Medial Eminence Line for Predicting Tibial Fracture Risk after Unicompartmental Knee Arthroplasty. Clin. Orthop. Surg..

[20] Watrinet J., Sandriesser S., Blum P., Augat P., Hollensteiner M., Schipp R., Fürmetz J., Reng W. (2025). Does undersizing of the tibial component in unicompartmental knee arthroplasty increase the risk of fracture? A biomechanical study. Knee Surg. Relat. Res..

[21] Kolessar D.J., Hayes D.S., Harding J.L., Rudraraju R.T., Graham J.H. (2022). Robotic-Arm Assisted Technology’s Impact on Knee Arthroplasty and Associated Healthcare Costs. J. Health Econ. Outcomes Res..

[22] Gordon A.M., Nian P., Mont M.A., Golub I. (2025). Comparison of implant complications, lengths of stay, and costs among patients undergoing robotic-assisted versus conventional unicompartmental knee arthroplasty. Knee.

[23] Guild G., Schwab J., Ross B.J., McConnell M.J., Najafi F., Bradbury T.L. (2025). Is Robotic-Assisted Unicompartmental Knee Arthroplasty Compared to Manual Unicompartmental Knee Arthroplasty Associated With Decreased Revision Rates? An Updated Matched Cohort Analysis. Arthroplast. Today.

